# Reducing the length of postnatal hospital stay: implications for cost and quality of care

**DOI:** 10.1186/s12913-015-1214-4

**Published:** 2016-01-15

**Authors:** John Bowers, Helen Cheyne

**Affiliations:** 1 Stirling Management School, University of Stirling, Stirling, FK9 4LA UK; 2 Nursing, Midwifery and Allied Health Professions Research Unit, Stirling University Innovation Park, Unit 13 Scion House, Stirling, FK9 4NF UK

**Keywords:** Postnatal care, Early hospital discharge, Length of stay, Cost savings, Care quality

## Abstract

**Background:**

UK health services are under pressure to make cost savings while maintaining quality of care. Typically reducing the length of time patients stay in hospital and increasing bed occupancy are advocated to achieve service efficiency. Around 800,000 women give birth in the UK each year making maternity care a high volume, high cost service. Although average length of stay on the postnatal ward has fallen substantially over the years there is pressure to make still further reductions. This paper explores and discusses the possible cost savings of further reductions in length of stay, the consequences for postnatal services in the community, and the impact on quality of care.

**Method:**

We draw on a range of pre-existing data sources including, national level routinely collected data, workforce planning data and data from national surveys of women’s experience. Simulation and a financial model were used to estimate excess demand, work intensity and bed occupancy to explore the quantitative, organisational consequences of reducing the length of stay. These data are discussed in relation to findings of national surveys to draw inferences about potential impacts on cost and quality of care.

**Discursive analysis:**

Reducing the length of time women spend in hospital after birth implies that staff and bed numbers can be reduced. However, the cost savings may be reduced if quality and access to services are maintained. Admission and discharge procedures are relatively fixed and involve high cost, trained staff time. Furthermore, it is important to retain a sufficient bed contingency capacity to ensure a reasonable level of service. If quality of care is maintained, staffing and bed capacity cannot be simply reduced proportionately: reducing average length of stay on a typical postnatal ward by six hours or 17 % would reduce costs by just 8 %. This might still be a significant saving over a high volume service however, earlier discharge results in more women and babies with significant care needs at home. Quality and safety of care would also require corresponding increases in community based postnatal care. Simply reducing staffing in proportion to the length of stay increases the workload for each staff member resulting in poorer quality of care and increased staff stress.

**Conclusions:**

Many policy debates, such as that about the length of postnatal hospital-stay, demand consideration of multiple dimensions. This paper demonstrates how diverse data sources and techniques can be integrated to provide a more holistic analysis. Our study suggests that while earlier discharge from the postnatal ward may achievable, it may not generate all of the anticipated cost savings. Some useful savings may be realised but if staff and bed capacity are simply reduced in proportion to the length of stay, care quality may be compromised.

## Background

Across the UK and in other countries with developed welfare states, health services face the twin challenges of increasing both quality and efficiency due to increased demand for services in a climate of financial constraint [1]. Healthcare needs and expectations are increasing, as people live longer but with more long-term chronic conditions. Health technologies and treatments have continued to advance offering the potential for significant health gains but at increased costs. At the same time health spending per person in the UK has not kept pace and is likely to remain flat in real terms over the next few years [1]. This means that the National Health Service (NHS) is under increasing pressure to make efficiency savings while improving the quality of services, essentially, to do more and better for less [2].

UK organisations such as Monitor [2] provide guidance and examples of ways in which cost savings might be achieved, including reductions in the length of hospital stay and increasing bed occupancy. However for anticipated savings to be realised this advice must be effectively adapted to the local context, considering the full range of impacts on cost and quality. Those tasked with local commissioning of services, often health service managers and clinicians must make judgements and decisions about service development and delivery in their own area, deciding which services will receive increased resources and in what areas savings and efficiencies can safely be made. Too often NHS service redesign and planning is based on intuitive judgements, incomplete evidence and data that is difficult to interpret.

In this paper we use an example from maternity care to examine the possible implications of reducing the length of hospital stay, a commonly advocated policy to increase service efficiency [2]. We explore and discuss the consequences for cost and quality of care, challenging commonly held beliefs about potential savings.

### Maternity care in the UK

Maternity care in the UK is a high volume universal service. Having a baby is the most common reason for UK hospital admission with around 800,000 births annually at a cost to the NHS of over £2.5 billion per year [3]. There are a number of socio demographic and clinical trends that suggest that costs of maternity care will continue to rise. Birth rates have increased; the average age at which women give birth is rising and more women with co-existing medical conditions are becoming pregnant resulting in increased numbers of women experiencing complex pregnancies and a corresponding increase in rates of maternal and infant morbidity [3].

Provision of high quality maternity care is essential for the health and wellbeing of mothers and babies. Complications of pregnancy and birth can have devastating and high profile consequences both for families and maternity service providers. Loss of care quality and in particular, perceived reduction in safety of care is unacceptable in a maternity service described as the ‘shop window of the NHS’ [4]. The challenge for maternity service managers is to determine which aspects of maternity services may be safely reconfigured or reduced to enable required cost savings without compromising safety and quality of care.

Midwives provide the majority of maternity care for healthy pregnant women in the UK with additional involvement from obstetric and medical teams for women with more complex obstetric and/ or medical complications. Care typically comprises three stages; antenatal care is provided to women from early pregnancy (around 10 weeks of pregnancy) through a recommended schedule of appointments (on average 8–10), intrapartum care, provided during labour and birth with around 98 % of women giving birth in a maternity hospital or community maternity unit, and postnatal care. After giving birth all UK mothers and babies receive midwifery care, first in hospital and then in the community (home or clinic) for a minimum of ten days (with around 3–6 visits) and for as long thereafter as necessary [5]. There is a widely held perception that postnatal care has long been a neglected aspect of maternity care and that it is an ‘easy target’ for cost saving initiatives [6–8]. Midwives report that staff and resources are often directed from postnatal care to antenatal clinics or labour ward and a recent survey conducted by the Royal College of Midwives found that 65 % of midwife respondents reported that organisational pressures were the key determinant of postnatal care planning rather than individual care needs of mothers and babies [9]. The status of postnatal care is further reflected in the current distribution of payments for services in NHS England where the large majority of funding under the Payment by Result scheme [4] is focussed on antenatal and intrapartum services. Under this scheme hospitals in England will receive only around £250 per mother/ baby for ‘standard’ postnatal care although actual care costs are more likely to be around £1000.

**Fig. 1:**
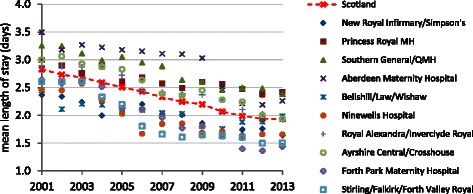
The trends in postnatal length of stay across Scotland

It is claimed that there has been an overall reduction in postnatal care services in the UK [9]. One key component of postnatal care is the hospital stay following birth; this has steadily decreased over the last two decades. In Scotland the mean postnatal hospital stay fell from 2.8 days in 2001 to 1.9 days in 2013 (Fig. 1), following the UK trend over the previous decade. In the late 1970s and 80s it was expected that women would remain in hospital for around 6 days [10]. By 1990 56 % of women in England remained in hospital for three or more days following birth [11]. Although there is considerable variation between UK hospitals’ mean length of stay, all have seen a substantial decline. In England almost 70 % of women now remain in hospital for less than two days after giving birth [12]. This may reflect service flexibility and woman’s choice – a move away from the previously very prescriptive practices. However, in recent years it appears that the trend towards shorter postnatal hospital stay, for women of all acuity levels (degree of health or social care need) has largely been driven by the need to improve service efficiency and reduce costs.

Comparisons between hospitals in Scotland (Fig. 1) may suggest that some areas have the potential to reduce average postnatal stay further. A lower demand for bed-days should reduce the staffing requirement and costs. However, a shorter length of postnatal stay may also impact on quality of care. In this paper we pose the question what is the impact on cost and quality of care of further reducing the length of postnatal stay? Specifically we discuss:

- the staff requirement; examining staff level and activity associated with the phases of the postnatal hospital stay.
- the bed requirement; given the variable, unpredictable nature of demand, 100 % utilisation is not consistent with the need to provide good access to postnatal beds and a contingency capacity is needed.
- quality of care within the hospital; many of the dimensions of quality are not readily quantified and must be interpreted in different care contexts. For postnatal care hospital readmissions and women’s experiences of care may provide some insight.
- women’s recovery and the consequences of early discharge for the community postnatal care service.

### Method

To address the above questions we draw on a range of pre-existing data sources including, the national maternity care experience surveys conducted in Scotland and England in 2013 [13–15] data from the Scottish Government Information Services Division [16], data routinely collected at NHS board level and the Nursing and Midwifery Workforce and Workload Planning (NMWWP) data [17]. Specific data sources are described at each section. Simulation [18, 19] and financial models were used to estimate excess demand, work intensity and bed occupancy to explore the quantitative, organisational consequences of reducing the length of stay. These various measures of efficiency data are considered in conjunction with the IOM quality domains [20] (safe, effective, equitable, timely, patient centred), to provide a holistic analysis of the potential impacts of further reductions in length of stay.

### Discursive analysis

#### Impact on cost

A major motivation in reducing length of hospital stay is to reduce costs, at least within the acute hospital. Some costs, such as those associated with the various support services and the whole hospital infrastructure, may be assumed proportionate to the length of hospital stay. However, other costs are directly attributable to the staff activities involved in a patient’s care and may not reduce directly in proportion to length of stay; a shorter length of stay could imply more intensive staff care but with relatively little reduction in the total input. Many studies of efforts to expedite discharge assume a simple cost for each day’s stay potentially exaggerating the potential for savings, though some [21, 22] do recognise the more subtle relationship between length of stay and costs.

### Staff requirement

The hospital stay may be considered as three phases, admission, recovery and discharge, each with different staff requirements. In many healthcare specialties the main costs are associated with the initial phase when there is more intensive activity, investigation and intervention, while the reductions in length of stay are typically achieved by shortening the lower dependency recovery phase [23]. In postnatal care the discharge phase also requires considerable staff input, preparing the mother and baby for their return home.

We used data from the Scottish Nursing and Midwifery Workload and Workforce Planning (NMWWP) project to examine maternity care staff activity for different phases of routine postnatal hospital care and develop a financial model to assess possible cost savings. The Scotland wide NMWWP project [17] aimed to inform more effective use of staff resources [24]. It involved considerable detailed data collection on five hospital labour wards, two postnatal wards and four community maternity units across Scotland. The NMWWP project recorded activity over 6272 staff-hours at 10 min intervals, distinguishing 89 different tasks. The hospital activity was recorded by trained observers while the community based activity was self-recorded by midwives providing care. In addition, the mother and baby requirements were noted for each activity observation, classified by the descriptions of acuity (Table 1). The NMWWP data also distinguished the maternity care staff by NHS pay bands. Within the UK, non-medical, maternity care staff are employed in grades (bands) based on level of education / professional registration. Bands 2 to 4 are maternity support workers who have a range of training and skills but are not professionally registered and work under the supervision of midwives. In some areas nursery nurses (trained in care of infants from 0-5years) are also employed at band 4. Band 5 are registered midwives (entry level), band 6 and 7 are midwife and midwife team leader respectively [25].

**Table 1:** NMWWP definitions of acuity [17]

Acuity	Example/ descriptors of mother and baby condition	Care required
0	Healthy term baby (37–42 weeks of pregnancy) with no risk factors. E.g. normal birth, mother’s age 16–40, BMI 18–35, number of previous births <5, requires routine care, mother able to care for baby independently	Routine
1a	Some obstetric or neonatal medical risks. E.g. urinary tract infection, diabetes, minor haemorrhage, BMI <18 or >35, low neonate temperature or slow feeding	Some increase in care.
1b	Some social risks. e.g. mother leaving ‘looked after’ services, smoker, criminal justice activity not related to child protection.	Some increase in care.
2	Medical or social risk factors requiring further intervention e.g. post caesarean section day 1 and 2, low birth weight <2.5 kg, child protection concerns.	Additional care often involving liaison with other services.
3	All mothers during labour and 2 hr after delivery. Mother who have experienced major obstetric complications e.g. haemorrhage. Puerperal psychosis, bereaved mothers.	Continuous one to one care.

A typical mother with no substantial complications might spend 36 hr in hospital after giving birth; the NMWWP data suggest that the mean actual care provided during this stay is 9.1 staff-hours. Table 2 summarises the distribution of the staff-time by activity and by phase of stay, distinguishing the direct care (face-to-face contact with the mother or baby) and indirect care (support related to a specific mother or baby). The associated care activities include housekeeping tasks and general administration: activities essential for the whole ward but not related to a specific mother. We asked four experienced senior midwives to independently attribute and apportion each of the NMWWP activities to the different phases of care. Some activities are clearly related to a single phase while others may be distributed across several phases. Their average estimates are included in Table 2. The overall distribution of activity on the postnatal ward suggests that 22.7 % or 2.1 hr of the staff input is associated with admission and 25.1 % or 2.3 staff-hours with discharge. With the remaining 52.2 % or 4.8 staff-hours dedicated to the recovery phase. Table 2 also provides a breakdown between phases of care for each activity. For example, on average a mother receives 0.1 hr of “parent education”, with most (53.9 %) being provided during the recovery phase of the postnatal stay.

**Table 2:** Distribution of staff time by activity and phase of hospital postnatal stay [17]

	Staff time		Phase of stay on ward			
	%	Hours	Admission	Recovery	Discharge	Band 2
Direct care						
communication	5.2 %	0.47	21.0 %	28.9 %	50.0 %	3.6 %
standard physical exam postnatal check	7.8 %	0.71	27.6 %	39.5 %	32.9 %	0.0 %
additional physical exam. e.g. catheter care/ wound care	7.2 %	0.66	39.2 %	39.3 %	21.4 %	5.3 %
personal care hygiene	5.5 %	0.50	59.7 %	24.5 %	15.7 %	32.4 %
administration of medicines	9.5 %	0.87	25.6 %	53.3 %	21.1 %	0.0 %
parent education	1.4 %	0.13	14.6 %	53.9 %	31.4 %	44.0 %
assessment (history & risk)	1.5 %	0.14	21.1 %	21.1 %	57.7 %	0.0 %
feeding advice & assistance	7.3 %	0.66	38.7 %	31.0 %	30.3 %	19.5 %
midwife procedure e.g. taking swabs	1.2 %	0.11	25.0 %	75.0 %	0.0 %	2.0 %
Indirect care						
documentation	18.4 %	1.68	31.8 %	26.9 %	41.3 %	1.4 %
liaison & referral	8.1 %	0.74	17.6 %	28.2 %	54.2 %	9.0 %
Associated activities (ward)	26.9 %	2.45	0.0 %	100.0 %	0.0 %	65.1 %
Total	100.0 %	9.11	22.7 %	52.2 %	25.1 %	22.9 %

**Fig. 2:**
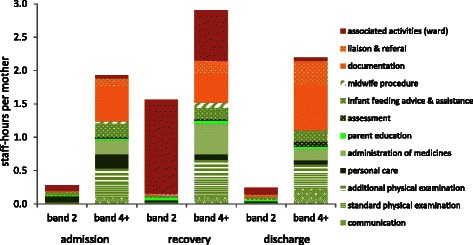
Maternity care staff input for major activities distinguishing admission, routine and discharge

Less qualified band 2 staff are used in several activities (Table 2). The staff mix varies but typically about 23 % of the staff-hours are provided by band 2 maternity care staff and 77 % by band 4, 5 and 6 staff (“band 4+”). Band 2 staff contribute relatively little to admission and discharge but they play a more prominent role in the recovery phase in particular undertaking personal care, parent education and advice and support as well as general housekeeping. The consequent distribution of the 9.1 hr of staff activity over the three phases, distinguishing the band 2 staff contribution, is illustrated in Fig. 2.

Assuming that the admission and discharge activities are essential, any reduction in length of stay is achieved by shortening the recovery period of care. However, the mean intensity of care during this phase is lower and much of the care is provided by the cheaper band 2 staff (in 2014 band 2 staff cost £23.10 per hour; band 4+ staff cost £44.69 per hour, weighted by the distribution of staff hours on the postnatal wards observed in the NMWWP exercise). Considering just the maternity care staff and excluding the other costs of providing a hospital bed (Table 3), the mean staff cost is £362 per mother. This includes the fixed costs of £93 for admission on to the postnatal ward and £104 for discharge, with £166 being associated with the recovery phase and proportionate to the length of stay. Table 3 distinguishes between staff grades: only a small proportion of the maternity staff costs, just £48 of £362, are related to band 2 staff though given their lower hourly rate this represents about 23 % of the total maternity staff time. In addition to the cost of maternity staff, a stay on the postnatal ward incurs charges due the occupancy of a bed with the associated hospital infrastructure, amounting to £330.

**Table 3:** The mean cost of a 36 hr postnatal stay distinguishing the phase of care and staff grade

	Hourly rate	Admission	Recovery	Discharge	Complete stay
band 2	£23	£7	£36	£6	£48
band >2	£45	£86	£130	£98	£314
all maternity care staff		£93	£166	£104	£362
bed	£9	£20	£287	£22	£330
total		£113	£454	£126	£692

The mean total cost of a mother’s 36 hr postnatal hospital stay is estimated to be £692, including the costs associated with the provision of a bed, as in Table 3. A simple estimate might suggest that shortening the mean stay by 17 % to 30 hr would reduce the costs of the maternity staff and bed-stay over the recovery phase proportionately reduced by 17 % to £577, as noted in Table 4. However, the tasks associated with admission and discharge require a fixed time, regardless of the length of stay. When these fixed costs of £113 for admission and £126 for discharge are recognised, the reduction in the bed-stay just affects the cost of the recovery phase, reducing this by 17 % from £454 to £378. Hence the total cost of the postnatal stay is £617.

**Table 4:** The potential cost savings of reducing postnatal length of stay

Scenario/assumptions	Mean length of stay (hours)	Cost per mother
Current	36	£692
reduced length of stay with proportionate costs	30	£577
reduced length of stay considering fixed costs of admission and discharge	30	£617
reduced length of stay, considering fixed costs & contingency capacity	30	£639

### Bed requirement – contingency capacity

Given the uncertain nature of much of the demand for postnatal care, 100 % bed utilisation is impossible and some contingency capacity is needed to ensure reasonable bed availability. It has been suggested that periodic bed crises can be expected if the mean bed occupancy is too high and that even a mean of 90 % will imply many instances of 100 % occupancy and recurrent problems with shortages of beds [18]. The contingency capacity should reflect the consequences of a bed shortage and the specialty’s bed demand characteristics. In particular, the contingency is more easily organised when the volume of patients is large. For example, a very large ward with a mean admission rate of 20 patients per day is very unlikely to experience admissions of 30, whereas a small ward with a daily mean of just 6 admissions will often have to cope with 9 or more: a contingency capacity of 50 % should ensure that the very large ward very rarely has problems with bed shortages whereas 50 % contingency is unlikely to be sufficient for the small ward. In general a smaller ward requires a proportionally greater contingency. Simulation [19] can provide insights into the trade-off between resource utilisation and the service level, as measured by bed shortages, and help identify the appropriate contingency capacity. A shorter mean length of stay should imply that a smaller ward will be sufficient; the appropriate number of beds can be determined using a simulation to examine the trade-off between bed occupancy and the probability of excess demand; the trade-off involves a consideration of the implications of excess demand.

**Fig. 3:**
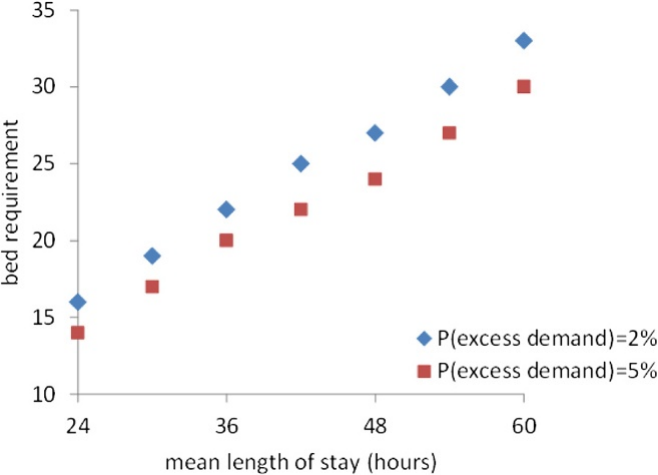
Bed requirements to provide a specified level of service

We constructed a simulation model of a typical postnatal ward with 3500 admissions per annum and used it to explore the relationship between the length of stay and the bed requirement. A series of simulation experiments was undertaken to identify the bed capacity needed to provide a specified level of service, measured as the probability that a postnatal bed is not available when needed by a mother, or P (excess demand). Two levels of service were considered with P (excess demand) = 2 % and 5 %. While undesirable, such an event might trigger various responses, such as expediting another mother’s discharge, without having any significant effect on patient safety. The simulation experiments were repeated with different mean lengths of stay, from 24 to 60 hr, and the results are summarised in Fig. 3. Assuming a mean length of stay of 36 hr and 3500 births p.a., 22 beds should be sufficient to ensure that a bed is immediately available for 98 % of admissions. If this length of stay could be shortened to 30 hr, 19 beds would provide a similar level of service. While fewer beds and staff would be required, their utilisation would fall implying an increase of 4 % in hourly staff and bed costs, implying a total mean cost of a mother’s postnatal stay of £639. As summarised in Table 4 examining the impact of a reduction in postnatal stay from 36 to 30 hr, or 17 %, a simple assumption that staff and beds can be reduced proportionately to the mean length of stay implies a cost of £577 or a saving of £115 per mother but if the staffing truly reflects the activity associated with admission and discharge, the saving is reduced to £75 or 11 %; if the contingency capacity is preserved the saving is reduced further to just £53 or 8 %.

### The impact on quality of care

The purpose of postnatal care is to support the physical and emotional recovery of the mother and baby, to promote parenting confidence and wellbeing and establish infant feeding in the first few days and weeks following birth. ‘High quality’ postnatal care must therefore succeed in achieving this purpose across each of the IOM quality dimensions [20]. While some indicators of quality may be objectively measured many can only be assessed with reference to patient experience. For example, safe care is that which avoids harm, in the postnatal care context this generally relates to detection and treatment of complications related to childbirth e.g. postnatal depression or sepsis, as well as avoidance of care related harm e.g. hospital acquired infection. Efficiency is largely assessed in relation to costs (discussed above). However, the remaining dimensions (including effectiveness) are often only assessed through women’s self-reported experience of care. For example, was infant feeding support provided when it was needed? Was information consistent and timely? Were women’s choices respected?

### Safe postnatal care

During postnatal care, physical assessments (e.g. monitoring of vital signs, wound checks) provide the opportunity to detect deviations from normal patterns of recovery. Table 2 indicates that most (around 60 %) of the physical examination and assessment of mother and baby, and infant feeding support and advice (around 70 %) is provided by trained maternity care staff during the admission and discharge phases of postnatal ward stay. We suggest that it seems likely therefore, that reducing the length of the recovery phase in hospital would not impact on the detection of complications assuming that there is ongoing monitoring and assessment by trained staff following hospital discharge.

Concern is often expressed that reducing the length of postnatal hospital stay increases the number of maternal and neonatal readmissions where complications arising in the early postnatal period are undetected, and this may be used as a proxy indicator of care that is not safe. We explored this using data in a limited study of discharge and readmission rates over a three year period gathered from one postnatal ward. Despite continuing reductions in the length of stay the readmission rate had also fallen, as noted in Table 5. Further studies are needed but our findings are consistent with the National Audit Office [3] report on maternity services in England which that found that while the readmission rate for babies had increased slightly (0.7 %) between 2008/9 and 2011/12 the rate for mothers was unchanged. A Cochrane Review of early postnatal discharge [26] also found (tentatively) no differences in maternal or neonatal readmissions. Readmissions to the maternity unit may provide a useful indicator of safe care although it underestimates the number of mothers and babies requiring additional clinical care but who are not readmitted to the maternity unit. A more comprehensive study might also consider consequent admissions to other wards and GP attendances.

**Table 5:** Postnatal ward readmission rates

Year	Mean length of postnatal stay (days)	Readmission rate
2010/11	2.07	2.9 %
2011/12	1.99	2.5 %
2012/13	1.94	2.5 %

### Women’s experience of care

**Fig. 4:**
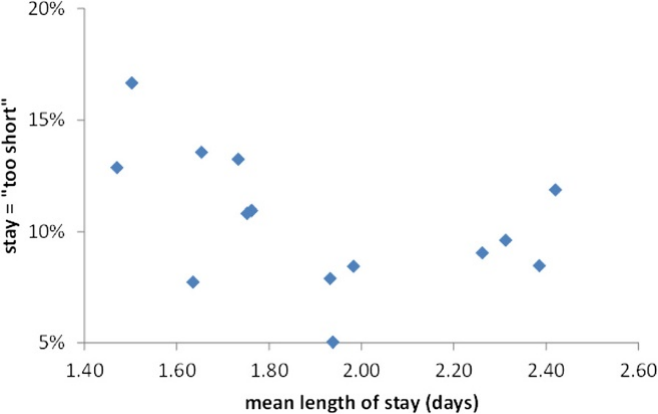
Dissatisfaction with the length of the postnatal stay

**Fig. 5:**
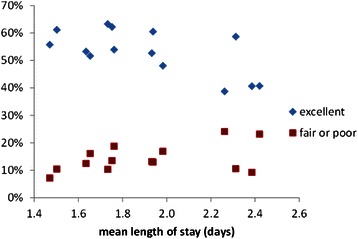
Overall experience of the maternity hospital and the length of the postnatal stay

**Fig. 6:**
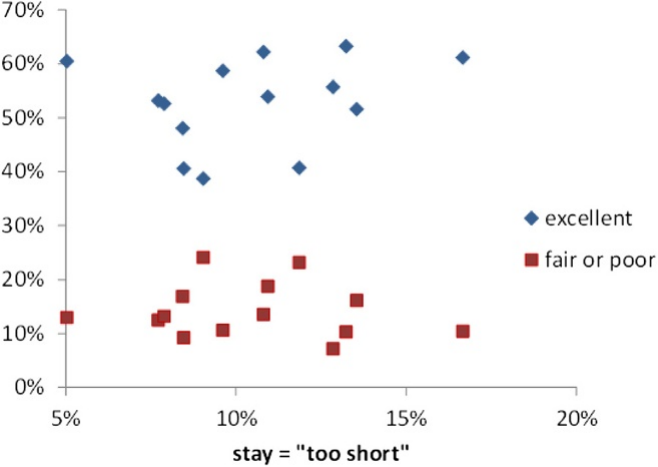
Overall experience of the maternity hospital and dissatisfaction with the length of the postnatal stay

Several recent UK surveys of mothers experience found that women were generally less satisfied with postnatal care than with either antenatal or intrapartum care [13, 14]. This is a reversal of the situation some years ago, at least in Scotland, when women were most satisfied with their postnatal care [27]. However, the postnatal length of stay does not, in itself, appear to be a major factor in mothers’ satisfaction with care. Studies of the relationship between length of stay and patient satisfaction in other specialties also suggest that there is no correlation [28]. We reanalysed summary data drawn from the NHS Board results of a national Scottish maternity care survey [15] for 13 major maternity hospitals with varying mean postnatal length of hospital stay (1.4 to 2.4 days). This revealed just a small negative correlation between the length of stay and mothers’ views that the stay is “too short” see Fig. 4. The two hospitals with the shortest mean stays have a relatively high proportion of mothers dissatisfied with their stay, approximately 13-17 % saying that their stay was “too short”. However, this specific criticism was not translated into overall dissatisfaction with care on the postnatal ward. Figure 5 suggests that there is little correlation between a hospital’s mean length of stay and the proportions of mothers rating their overall experience as excellent, or fair-poor, Fig. 6 compares the proportions of mothers stating that their stay was too short with their overall experience. There is no discernible correlation. Although there is substantial variation in mothers’ satisfaction with postnatal care in hospitals it appears that factors other than the length of stay are more important.

**Fig. 7:**
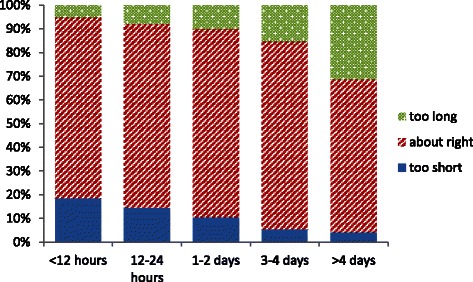
Mothers’ satisfaction with their length of stay

Figures 4, 5, 6 illustrate the summary statistics for each hospital. When the individual responses were examined, some of the variation in mothers’ experiences and preferences were revealed. Figure 7 suggests that although 18 % of mothers having a stay of less than 12 hr described it as “too short” but 5 % thought even this short stay was “too long”. As the length of stay increases, so more mothers express concern that it is too long but even a stay of >4 days is judged “too short” by some mothers. Some mothers prefer a shorter stay and some a longer stay; ideally mothers should be offered some choice in their length of postnatal stay as having individual choice may be more important than the actual length of stay.

Women’s experience of the content and delivery of care while in hospital may be a more important indicator of quality than the length of hospital stay and is likely to contribute to whether women feel their length of stay is too long or too short. The national surveys of women’s experience of maternity care undertaken in Scotland and England in 2013, using the same questionnaire [13, 14] found that considering postnatal care in hospital, around 40 % of women did not get the information they required, only around 60 % of women received active support and encouragement from staff about feeding their baby and around one third said they were only sometimes, or were not, treated with kindness and understanding. To what extent might these aspects be affected by reduction in length of stay and increasing bed occupancy? Data from the NMWWP (Table 2) suggests that in the postnatal ward, very little staff time is spent on communication with mothers, parent education, and feeding advice and assistance (only around 75 min per mother in total). The majority of general communication and feeding advice and assistance (around 70 %) appear to be associated with admission and discharge with only about 30 % (or 20 min) taking place through the recovery phase. It seems that reducing the hospital stay would therefore have little impact if the staffing is maintained to complete the fixed admission and discharge activities. However, only around 8 min of staff time was spent on parent education, half during the recovery phase. Shortening the length of hospital stay could mean that even less parent education would take place during the hospital stay.

Women’s experience of receiving general support and kindness relate to the way in which all interactions between mothers and staff take place rather than to specific tasks. These aspects may be adversely affected by reduction of length of stay and increasing bed occupancy. The nature of care varies over the postnatal stay, with much less intensive care during the recovery phase. A postnatal ward with a shorter mean length of stay is inevitably busier with more staff activity per bed. When estimating the cost savings (Table 4), it was assumed that the staffing reflects the need for more intensive care during the admission an discharge phases. If staff numbers are simply reduced in proportion to the bed-days requirement, individual workloads will increase, with possible implications for the quality of care as staff work under increased time pressure [29]. Further, Fig. 2 indicates that during the recovery phase staff activity is more directed to some non patient-related tasks and staff are able to take their breaks. Reducing time staff may spend away from the ‘emotion work’ of direct care and increasing the intensity of their work load may reduce staff’s ability to care for women in a compassionate manner.

### Recovery during hospital stay and changing acuity

**Fig. 8:**
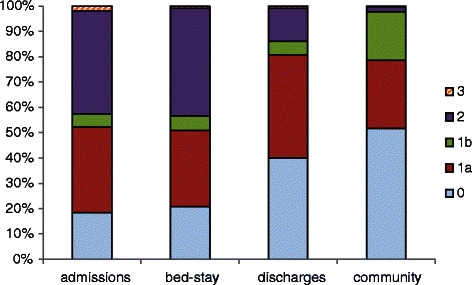
Maternity acuity category (ward A)

**Fig. 9:**
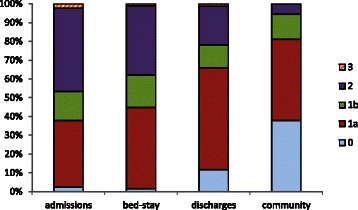
Maternity acuity category (ward B)

We found some evidence that time spent in hospital following birth does provide the opportunity for recovery. The development of the NMWWP tool involved collecting data describing both staff activity and the mothers’ acuity, so that a unit’s staffing might reflect its profile of mothers’ requirements. The acuity, or dependency, of each mother/baby was categorised as the staff activity was recorded, using the definitions of Table 1. As mothers move through postnatal care recovering from labour and birth, so their acuity tends to decline, as illustrated in Figs. 8, 9. Using data from the NMWWP two postnatal wards with very different mean lengths of stay were examined, ward A had a mean stay of 55 hr and ward B, 27 hr. On admission to the postnatal ward A 42 % of mothers/ babies were in acuity category 2 or 3 but this declined to 14 % on discharge hence 67 % of the mothers/ babies categorised as category 2 or 3 were discharged with an acuity of 0 or 1. Ward B had a slightly different profile on admission, with proportionately fewer acuity 0 mothers/ babies and 47 % in category 2 or 3, declining to 22 % on discharge: just 53 % of the mothers/ babies categorised as category 2 or 3 on admission to ward B are discharged with an acuity of 0 or 1. Compared to ward A, ward B mothers have a shorter stay and that may be the cause of more mothers/ babies being discharged with higher acuities.

### Consequences of shorter lengths of stay for the community postnatal care service

Although mothers’ acuity declines over the hospital postnatal stay, the acuity levels on discharge indicate that community postnatal care is vital. Significant numbers of women are classed as category 2 at discharge from hospital – these are women recovering from caesarean section, low birth weight babies or babies with feeding problems, women with mental health problems and women with significant social problems. Around half (46 % on ward A and 66 % on ward B) of women were categorised as 1a or 1b at discharge, these are mothers and babies who are deemed to require significant clinical and or social support in the postnatal period. While reductions in postnatal stay may be achievable, and even desirable, maternity care in the community should then be correspondingly enhanced to meet the physical and psychosocial needs of mothers and babies [30] and maintain care quality. However, as length of postnatal stay has been reduced in the UK there is evidence that community based maternity care has also been reduced some areas. Successive national maternity surveys conducted in England have shown an increase in the proportion of women receiving only one or two midwife visits following hospital discharge from 15 % in 2007 to 25 % in 2013. Reducing both length of stay and community maternity services is likely to pose a risk of compromise to care quality for mothers and babies.

## Discussion and conclusions

In this paper we have drawn on a range of data sources and analytical techniques to explore the implications of reducing the length of postnatal hospital stay on cost and quality of care. This analysis and discussion will be of relevance to health policy makers and service providers in maternity care and many wider health care contexts. Healthcare policy makers and providers in the UK and in other countries with developed welfare states are currently challenged to reduce the cost of health care without compromising on health care quality. In this situation it is difficult for those charged with planning services to make well -informed decisions about how and where to target resources. This paper aims to both challenge and support those charged with making such decisions both in maternity care and in healthcare more generally. We have demonstrated that a wide range of consequences may be associated with even superficially relatively simple changes to healthcare provision and that intuitively cost saving enterprises may not realise anticipated returns if quality is to be maintained. However, we propose that use of existing readily available data, simulation and financial modelling provides a useful method of exploring such complex healthcare problems. This approach could be used to address other areas where services reconfiguration is considered and a range of stakeholder objectives and values must be integrated. The IOM quality domains tailored to context can provide a framework for identifying values and priorities and developing a more complete understanding of the consequences of policy changes.

In most developed countries with a welfare system, maternity care is a universal service accounting for a significant portion of national health care budgets. While antenatal and intrapartum services are generally recognised as high priority, postnatal care may be perceived as less critical and therefore a target for service economies. The wisdom of this view is questionable as while few mothers in high income countries die as a result of pregnancy and birth, the majority of those who die do so in the postnatal period [31]. Further, the increasing rate of caesarean section globally means that around one third of women are recovering from surgery at the same time as coping with the demands of a new baby. Despite this, the length of postnatal hospital stay has steadily fallen in the UK and in other countries, largely motivated by a desire for cost savings. This has raised concerns about the safety of mothers and babies [32, 33], in particular in areas where community postnatal care has also been reduced and in countries (such as Sweden [30] and Ireland) where community care is not routinely provided.

Some significant cost savings may be achievable by reducing the length of stay; however, the savings are not directly proportionate to the reduction in length of stay and may be considerably less than might be expected, if sufficient staffing is maintained to ensure reasonable quality of care. Otherwise the savings come at a cost both to quality of care and to the wellbeing of both mothers and babies and care staff. Several studies have reported that a significant minority of women report dissatisfaction with postnatal care [6, 13, 14]. In particular, women report receiving conflicting advice and that staff are often too busy to provide the information, support and care that they require. Data presented in this paper indicates that very little time during the postnatal hospital stay is currently given to communication with mothers, parent education and advice or infant feeding support. If staffing is simply reduced in proportion to the length of stay, the work intensity will increase giving staff even less time for activities that may be seen as non-essential for safe care yet are essential to achieve key aspects of quality of postnatal care. Further, as work load intensity increases staff spend proportionately more of their time on admission and discharge procedures and have less ‘down time’ and this could have substantial implications for staff wellbeing as well as quality of care [29].

Our analysis does not suggest that safety of care would be directly compromised by reducing length of stay for many healthy mothers and babies with low levels of acuity. Much of trained staff time is directed to assessment of mother and baby during admission and discharge procedures and readmission rates do not appear to be affected by marginal reductions in length of stay. However, this proposition is based on the availability of skilled midwifery care in the community to ensure that the safety and wellbeing of mothers and babies is maintained.

Our findings suggest that a longer hospital stay may benefit the higher acuity mothers/ babies as acuity typically falls over the course of a longer hospital stay. Merely discharging mother and babies earlier does not in itself reduce the time they require to recover from birth and to establish parenting skill and confidence. Therefore early discharge for the higher acuity mothers is likely to place greater demands on community postnatal care and provide poorer quality of care for this group of mothers.

### Assumptions and limitations

The data used for this analysis were collected for other purposes and have been adopted to explore the questions we posed. Some data sets used were small and local and therefore assumptions are more tentative, however, others such as the national surveys are likely to represent the experiences of postnatal women in the UK. The analyses assume that staff activity and mother/ baby acuity data are a reflection of current practice, and are representative of postnatal wards in general.

### Recommendations

Our findings may present those charged with reducing health care costs with a conundrum, indicating that reducing length of stay may not provide all of the anticipated cost savings and could have negative consequences for mothers and staff. However, we suggest that it is possible to achieve cost savings and maintain or improve quality by targeting resources appropriately. All mother and babies require skilled postnatal care however, not all require the same care. The traditional one size fits all approach should be abandoned with care pathways tailored to acuity level. Planning for postnatal care could begin during the antenatal period. Providing some continuity of care between antenatal and postnatal care would facilitate this and reduce repetitious and time consuming assessments. Skilled assessment of postnatal acuity level should be undertaken following birth with immediate (flexible) allocation to care pathway. The lowest acuity mothers may be discharged home directly from the labour suite thereby reducing workload in the postnatal ward areas. However, mothers and babies with higher level acuity should be given the opportunity to remain in hospital for longer giving time for recovery. Mothers preferred length of stay should be incorporated into care planning. Some of the cost saving achieved by reducing length of stay for mothers/ babies with low levels of acuity could be reinvested in enhanced postnatal care, focussed on particular groups (partly to reflect the higher acuity that can be associated with earlier discharge). Finally a team approach could be adopted both for ward and community based care with midwives leading a team comprising lower band staff supporting more care contacts but involving less costly staff. Length of stay itself is not critical to good quality care: staffing is more important. More targeted deployment of resources to reflect different mothers’ needs might even enable both cost savings and better quality of care.
